# The Use of a Nutrient Quality Score is Effective to Assess the Overall Nutritional Value of Three *Brassica* Microgreens

**DOI:** 10.3390/foods9091226

**Published:** 2020-09-02

**Authors:** Massimiliano Renna, Anna Maria Stellacci, Filomena Corbo, Pietro Santamaria

**Affiliations:** 1 Institute of Sciences of Food Production, National Research Council of Italy, via Amendola 122/O, 70126 Bari, Italy; 2 Department of Soil Plant and Food Sciences, University of Bari Aldo Moro, via Amendola 165/A, 70126 Bari, Italy; 3 Department of Pharmacy-Drug Sciences, University of Bari Aldo Moro, via Orabona, 4, 70125 Bari, Italy; filomena.corbo@uniba.it; 4 Department of Agricultural and Environmental Science, University of Bari Aldo Moro, via Amendola 165/A, 70126 Bari, Italy; pietro.santamaria@uniba.it

**Keywords:** *B. oleracea* var. *botrytis*, *B. oleracea* var. *italica*, *Brassica rapa* L. subsp. *sylvestris* L. Janch. var. *esculenta* Hort, Dietary Reference Values, hydroponics, mineral elements, nutrient solution, Principal Component Analysis, vitamins

## Abstract

Microgreens have immense potential for improving dietary patterns, but little information is available regarding their overall nutritional value. We evaluated the nutritional traits of three hydroponically grown *Brassica* microgreens by using a Nutrient Quality Score. Micro cauliflower, micro broccoli and micro broccoli raab were grown using nutrient solutions with three different NH_4_:NO_3_ molar ratios (5:95, 15:85, and 25:75). Protein, dietary fiber, β-carotene, α-tocopherol and mineral elements (Ca, K, Mg, Fe, Zn, Cu, Mn, and Na) were analyzed. We developed the Nutrient Quality Score (NQS 11.1) on the basis of 11 desirable nutrients and 1 nutrient (sodium) to be limited. All *Brassica* microgreens are an excellent source of Vitamins A and E (more than 20% of the daily reference value—DRV), as well as a good source of calcium and manganese (10–19% of the DRV). Micro cauliflower showed a NQS 11.1 at 47% higher than micro broccoli raab and micro broccoli. Using NH_4_:NO_3_ 25:75 molar ratio, the average score was 27% higher than other molar ratios. In all cases, the microgreens in the present study showed a higher NQS 11.1 than their mature counterpart (on the basis of data from the United States Department of Agriculture), highlighting that the score of micro cauliflower was about six-fold higher than mature cauliflower. In conclusion, the NQS 11.1 was useful for assessing the overall nutritional quality of the three *Brassica* microgreens, instead of simply quantifying nutrient content, in order to compare a single nutrient among different genotypes. Furthermore, the results highlight that the micro broccoli raab, micro broccoli and micro cauliflower in this study can be considered nutrient-rich vegetables that are able to improve dietary patterns more effectively than their mature counterparts.

## 1. Introduction

It is well known that an inadequate intake of some nutrients can be associated with hidden hunger [1], although the signs of this form of undernutrition may not be obvious in people affected by it. For both young people and older adults, hidden hunger can be due to an inadequate intake of one or more nutrients, including macro- and micro-nutrients [2]. For example, an adequate intake of dietary fiber may be useful for preventing constipation and reducing the risk of colon cancer and diseases associated with glucose metabolism [3]. Vitamin A deficiency is associated with some ocular diseases as well as with an increase in infectious morbidity and mortality in children, especially in low-income countries [4]. An adequate intake of α-tocopherol, the most common and biologically active form of vitamin E, can reduce the risk of degenerative diseases regarding the nervous system and muscles [5]. Regarding mineral elements, their key role should be considered in several functions, including the fact that their inadequate intake is inversely associated with good health, especially in older adults. For example, a calcium deficiency may cause osteoporosis and, therefore, an increased risk of bone fracture [6]. Likewise, magnesium also seems to positively affect bone health since it represents an important factor for osteoblast activity [7]. An adequate intake of zinc is particularly important in the elderly since the immune function decreases with age; thus, even a slight zinc deficiency in older adults can increase their susceptibility to infections [7]. Furthermore, other mineral elements, such as potassium, iron, copper and manganese, are involved in several functions. Therefore, an adequate intake of all these mineral elements should be considered essential for fighting hidden hunger, especially in older adults.

Microgreens can be considered as a functional food due to their high content in several healthy compounds for humans [8,9,10,11,12,13,14,15]. This emerging category of vegetable products can be defined as immature greens, which are harvested when the cotyledon leaves have fully developed and the first true leaves have emerged, although the term ‘microgreens’ lacks any legal definition [16]. It is important to note that the duration of the growing cycle of microgreens is intermediate between sprouts and “baby leaf” vegetables. Thanks to their flexibility in cultivation, several growing techniques have been applied to microgreens in order to modulate the content of nutritional compounds to obtain tailored food products [13,17,18,19,20,21].

Consumer interest in microgreens is increasing not only due to the well-known nutritional traits but also because of their gastronomic potential. It is possible to effectively produce microgreens by using seeds from several species and genotypes, providing a wide range of colors and shapes as well as a flavor that can be considered stronger in comparison to mature vegetables [16]. Therefore, microgreens are now gaining interest as garnishing greens and ingredients in a wide range of dishes, considering that some of their culinary applications include appetizers, main courses, desserts and smoothies [14,16]. Moreover, microgreens can be considered as an excellent vegetable resource for culinary applications and, at the same time, as a food product that is able to improve the intake of some nutrients.

Although several studies have been carried out regarding the nutritional evaluation of microgreens [8,9,11,13,15,22,23,24], only one reports the nutritional ranking of some microgreens on the basis of an index that is able to summarize the overall nutritional quality [14]. At the same time, the literature lacks research into the adoption of this evaluation method to be used not only to compare different genotypes but also to assess the effects of growing conditions on the microgreens’ nutritional quality. Therefore, the aim of this study is to assess the nutritional value of three hydroponically grown *Brassica* microgreens with different nutrient solutions by using a Nutrient Quality Score that includes the relative dietetic contribution of some macro- and micro-nutrients.

## 2. Materials and Methods

### 2.1. Cultivation of Microgreens and Sample Preparation

The project was carried out from April to May 2015 in a greenhouse at the Experimental Farm ‘La Noria’, as part of the Institute of Sciences of Food Production, Italian National Research Council, located in Mola di Bari (BA), Southern Italy (41°03′ N, 17°04′ E). The experimental factors were: (i) three genotypes and (ii) three NH_4_:NO_3_ molar ratios in the nutrient solution (NS).

**Figure 1:**
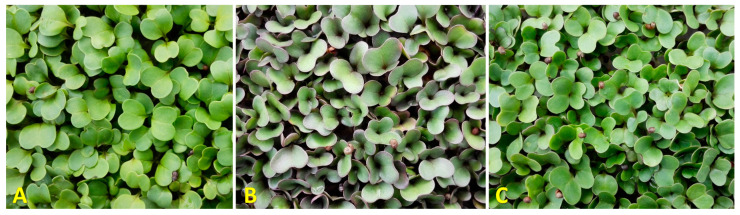
*Brassica* microgreens from the project: broccoli raab (**A**); cauliflower (**B**); broccoli (**C**).

The three genotypes of *Brassica* microgreens were broccoli raab (*Brassica rapa* L. subsp. *sylvestris* L. Janch. var. *esculenta* Hort), broccoli (*Brassica oleracea* L. var. *italica*) and cauliflower (*Brassica oleracea* L. var. *botrytis*) (Figure 1). For broccoli raab, the seeds of the local ‘Novantina’ variety were used, while for broccoli and cauliflower, the cultivars ‘Broccolo natalino’ and ‘Cavolfiore violetto’, respectively, were used. All seeds were purchased from the Riccardo Larosa Company (Andria, Italy). A hydroponic system, with polyethylene terephthalate fiber pads (40 cm × 24 cm × 0.89 cm; Sure to Grow^®^; Sure to Grow, Beachwood, OH, USA) as the growing medium, was used to grow the microgreens. The fiber pads were placed on a 2.7 m^2^ (1.0 m × 2.7 m) aluminum bench. All three genotypes were sown using the same density of 4 seeds cm^−2^. During germination, the growing media was irrigated manually and covered with black polyethylene film until the germination was complete.

The seedlings were fertigated by subirrigation with a nutrient solution (NS) type-like Hoagland and Arnon [25], having three different NH_4_:NO_3_ ratios (%): 5:95, 15:85 and 25:75. The element concentrations, expressed in mg L^−1^, were as follows: N 105, P 16, K 117, Ca 86, Mg 24. The electrical conductivity of the NS was between 1.12 and 1.42 mS cm^−1^, while the pH was between 5.8 and 6.3. The NS was distributed by a drip tape line with pressure-compensated drippers (each with a delivery rate of 0.133 L min^−1^), applying an open cycle management. The experimental design used was a split-plot, where each plot was represented by the nutrient solution (NS) located in the bench and each sub-plot was represented by a genotype.

Environmental conditions inside the greenhouse were measured using a CS21 5-L temperature and relative humidity probe (Campbell Scientific, Logan, UT, USA) and LI-190R quantum sensor (LI-COR, Lincoln, NE, USA), both connected to a CR1000 datalogger (Campbell Scientific, Logan, UT, USA). The average daily mean, minimum and maximum air temperatures inside the greenhouse during the experiment were 21.2, 15.4 and 29.1 °C, respectively. The average daily mean, relative minimum and maximum air humidity values were 55%, 31% and 84% respectively. The daily light integral (DLI) during the experiment ranged from about 5 to 19, with a mean value of 15 mol∙m^−2^∙d^−1^.

Harvesting was carried out when the first true leaves appeared by cutting the microgreens just above the surface of the growing media. Three samples were considered for each experimental unit (genotype and treatment), and analyzed as independent replicates. The harvested microgreens were freeze-dried (ScanVac CoolSafe 55-9 Pro; LaboGene ApS, Lynge, Denmark) to be used for chemical analysis.

### 2.2. Determination of Protein and Dietary Fiber

The protein content (N × 6.25) was determined by Kjeldahl nitrogen, according to the Association of Official Agricultural Chemists (AOAC) method 976.05 [26], and results were expressed as g 100 g^−1^ fresh weight (FW). The dietary fiber content was determined by the enzymatic-gravimetric procedure, according to the AOAC method [26] with some modifications reported by Palmitessa et al. [27]. Samples (250 mg) were incubated in 32.5 mL of H_2_SO_4_ 0.64 N for 10 min at 100 °C, adding a few drops of n-octanol as an antifoam agent. The mixture was filtered then washed with warm distilled water, and incubated in 32.5 mL of KOH 0.56 N for 10 min at 100 °C. Samples were then filtered and washed three times with acetone RPE and dried at 105 °C for one hour. The dried residue was subjected to ash determination by weighing the residue before and after the treatment in a muffle furnace at 550 °C for 3 h. Weight loss, corresponding to the dietary fiber, and results were expressed as g 100 g^−1^ FW. All chemicals used were supplied by Sigma-Aldrich (Milan, Italy) and were of analytical grade.

### 2.3. Determination of α-Tocopherol and β-Carotene

α-Tocopherol and β-carotene were extracted using the procedure described by Palmitessa et al. [27]. Quantification of the two lipophilic compounds was carried out with high-performance liquid chromatography (HPLC) by using an Agilent Technologies HPLC system, model 1100 (Agilent Technologies, Palo Alto, CA, USA) equipped with a quaternary pump solvent delivery, thermostatic column compartment and diode array detector (DAD) (Agilent Technologies, Palo Alto, CA, USA). According to Xiao et al. [8], acetonitrile/ethanol (50:50 *v*/*v*) was used for the isocratic elution of α-tocopherol and β-carotene into a reversed stationary phase ZORBAX EC18 (Agilent Technologies) (5 μm, 4.6 × 150 mm). Stop time was set at 30 min with a re-equilibration time of 10 min corresponding to the ~20 column volume (Vc = 0.52 mL). The column temperature was not controlled, while the flow was maintained at 1.2 mL min^−1^. Absorbance was measured at 290 and 450 nm respectively for α-tocopherol and β-carotene. Samples were run in triplicates, and quantification was completed based on calibration curves with 5 concentration points for each compound that was prepared separately. Calibration was performed by linear regression of peak-area ratios of the vitamins to the internal standard (β-apo-8′-carotenal) versus the respective standard concentration, obtaining R^2^ values of 0.9992 and 0.9999 for β-carotene and α-tocopherol, respectively. Results were expressed as mg 100 g^−1^ FW. All chemicals used were supplied by Sigma-Aldrich (Milan, Italy) and were of analytical grade.

### 2.4. Elemental Analysis

Freeze-dried plant tissue was digested with 2 mL 30% H_2_O_2_ and 8 mL 65% HNO3 for trace analysis, using microwave assisted pressure digestion. Total Ca, Mg, K, Na, Fe, Cu, Mn and Zn were determined in the extracts by Inductively Coupled Plasma-Optical Emission spectrometry (ICP-OES) [28,29].

### 2.5. Development and Calculation of Nutrient Quality Score

The calculation of the Nutrient Quality Score (NQS) was composed of several steps, according to Ghoora et al. [14] with some modifications. First, the daily intake (DI) of each nutrient was calculated per serving size of microgreens starting from the content of nutrients determined by the chemical analysis carried out in this study. We considered a serving size of 85 g taking into account the reference amount customarily consumed (RACC) that is specified for fresh vegetables [30] since a specific RAAC for microgreens is lacking, and also considering that the biological and physiological traits of microgreens can be considered as similar to their mature counterparts [31]. The equation used to calculate DI is reported below:

DI (mg day^−1^) = [nutrient content (mg 100 g^−1^)/100] × serving size (85 g) (1)

Subsequently, the percent of nutritional contribution (NC) of each nutrient per serving size of microgreens was calculated based on the Dietary Reference Values (DRV) reported in Table 1. The equation used to calculate NC is reported below:

NC (%) = [DI (mg day^−1^)/DRV (mg day^−1^)] × 100 (2)

The NQS 11.1 was calculated by adding the NC for 11 nutrients to be encouraged (protein, dietary fiber, vitamins A and E, Ca, Mg, K, Mn, Fe, Cu and Zn), then subtracting the NC of sodium since it is a nutrient to be limited. The equation used to calculate NQS 11.1 is reported below:

NQS 11.1 = Σ_1–11_ (NC_i_) − NC_Na_ (3)

The same method was also used to calculate the NQS 11.1 for mature broccoli raab, broccoli and cauliflower. In this case, the DI of each nutrient, per serving size, was calculated by referring to data from the United State Department of Agriculture [32,33,34].

### 2.6. Statistical Analysis

The data were analyzed by a two-way analysis of variance (ANOVA), using the general linear model procedure for SAS software (SAS Version 9.1, SAS Institute, Cary, NC, USA) and applying a split-plot design with genotype (G) as the sub-plot factor and the nutrient solution (NS) as the main factor for all measurements. All the means were compared by using the Least Significant Difference (LSD) test at *p* = 0.05; the standard deviation (SD) was also calculated.

The principal component analysis (PCA) was performed by using the PRINCOMP procedure (SAS software, Cary, NC, USA). The analysis was carried out on the set of 12 variables included in the computation of the Nutrient Quality Score (protein, fiber, β-carotene, α-tocopherol, Ca, K, Mg, Na, Zn, Fe, Cu and Mn). The PCA was carried out on the correlation matrix of the studied variables. Loadings were examined to identify the variables that most contributed to each selected PC.

## 3. Results

### 3.1. Nutrient Content in Microgreens

By using a nutrient solution with a NH_4_:NO_3_ 25:75 molar ratio, the highest protein content was found to be in micro cauliflower, while in all other cases the average protein content was 2.37 g 100 g^−1^ FW, without significant differences among the genotypes and NH_4_:NO_3_ molar ratios (Table 2). As regards the content of fiber, β-carotene and α-tocopherol, the interaction between genotypes and NH_4_:NO_3_ molar ratio was not significant; the average content of fiber, β-carotene and α-tocopherol was, respectively, 0.55 g 100 g^−1^ FW, 5.07 and 6.15 mg 100 g^−1^ FW (Table 2).

As for the mineral elements, the content of magnesium was highest in micro cauliflower that was grown using a NH_4_:NO_3_ 25:75 molar ratio, which translates into a content that was 47% higher than all the other cases (Table 3). By using a NH4:NO3 25:75 molar ratio, micro cauliflower and micro broccoli raab showed, respectively, the highest and lowest sodium content. The iron content in micro cauliflower that was grown using the NH_4_:NO_3_ 25:75 molar ratio was 46% higher than micro broccoli raab and micro broccoli grown using a NH_4_:NO_3_ 25:75 molar ratio, as well as micro broccoli grown using a NH_4_:NO_3_ 5:95 molar ratio and micro cauliflower grown using a NH_4_:NO_3_ 15:85 molar ratio (Table 3). The average content of calcium, potassium, zinc, copper and manganese were, respectively, 166 and 250 mg 100 g^−1^ FW, and 1310, 27 and 452 μg 100 g^−1^ FW, without any differences among genotypes or NH_4_:NO_3_ 25:75 molar ratios (Table 3).

### 3.2. Principal Component Analysis

Principal component analysis allowed us to summarize the main behaviors outlined with the univariate analysis of variance, highlighting the effect of the three Brassica genotypes and different nutrient solution molar ratios on nutritional parameters.

**Figure 2:**
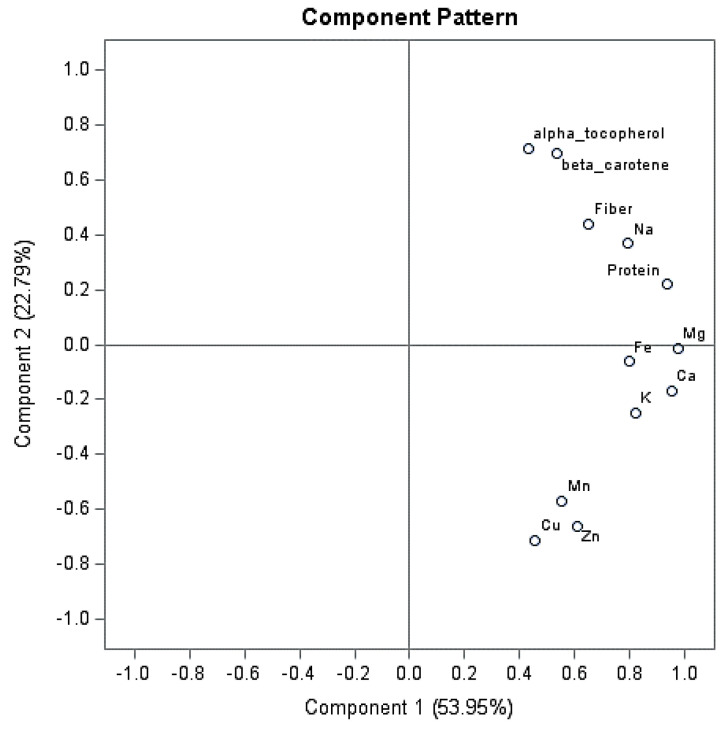
Loading plot for principal components 1 and 2, describing variation in nutritional parameters of three *Brassica* genotypes of microgreens grown using three NH_4_:NO_3_ molar ratios in the nutrient solution.

In detail, the first three principal components explained 83.2% of the total variance, with principal component 1 (PC1) and principal component 2 (PC2) accounting for 53.95% and 22.79%, respectively (Figure 2). PC1 was positively correlated with all parameters, with the highest weights observed for magnesium and calcium, followed by potassium, sodium and iron; in addition, protein content showed a high contribution to this PC. On the second PC, α-tocopherol and β-carotene weighted highly and positively, whereas copper, zinc and manganese showed high negative weights (Figure 2).

The inspection of the score plot for the first two components showed that the first PC clearly discriminated micro cauliflower grown using NH_4_:NO_3_ 25:75 molar ratio from the other treatments, thus highlighting the interactive effect of genotype and nutrient solution composition on plant nutritional properties.

**Figure 3:**
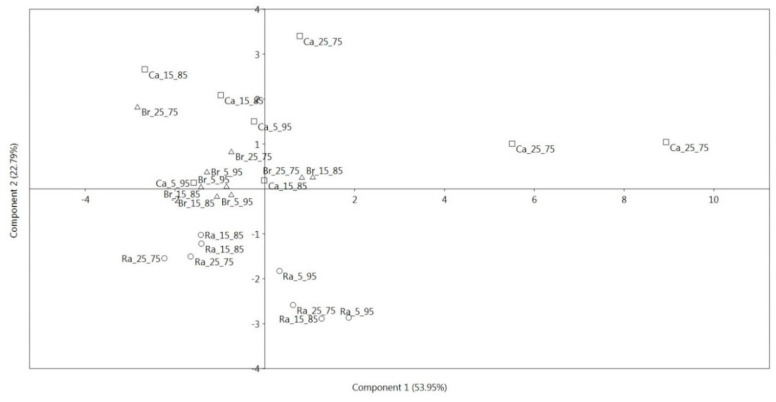
Score plot for principal components 1 and 2, describing variation in nutritional parameters of three *Brassica* genotypes of microgreens grown using three NH_4_:NO_3_ molar ratios in the nutrient solution. For each genotype, 5_95, 15_85 and 25_75 indicate, respectively, the NH_4_:NO_3_ molar ratios of 5:95, 15:85 and 25:75. Ra, micro broccoli raab; Br, micro broccoli; Ca, micro cauliflower.

This finding confirmed the results of univariate analysis of variance, which showed significantly higher contents of magnesium, sodium and iron for this treatment, whereas, on the other side, lower average values for micro broccoli raab (sodium and iron) and micro broccoli (iron) grown using the same molar ratio (Figure 3).

The second PC was able to underline the different overall behavior of the three genotypes, with particular regard to micro broccoli raab, which showed on average the largest copper, manganese and zinc contents, but the lowest average contents of α-tocopherol and β-carotene (Figure 3). A greater overlapping was observed for micro broccoli and micro cauliflower response (Figure 3), although average higher values of α-tocopherol and β-carotene, as well as a trend towards their increase passing from to 5:95 to 25:75 molar ratios, were observed for micro cauliflower. Finally, a clustering of observations for both micro cauliflower and micro broccoli grown under the 5:95 molar ratio was recorded, indicating an average content of α-tocopherol and β-carotene as well as copper, manganese and zinc for these factor level combinations (Figure 3).

### 3.3. Percent of Nutritional Contribution and Nutrient Quality Score

A serving size (85 g) of micro cauliflower grown using a NH_4_:NO_3_ 25:75 molar ratio provided a contribution of protein of almost 6% of the Dietary Reference Value, while in all other cases the contribution of protein was about 3.0–3.5% of the DRV (Table 4). The contribution of fiber ranged from 1.5% to 3.3% of the DRV, while regarding vitamins, the percent of nutritional contribution was considerably higher and variable. Effectively, the contribution of vitamin A (as β-carotene) ranged from 31% to 86% of the DRV, while vitamin E (as α-tocopherol) ranged from 12% to 98% (Table 4).

A serving size of micro cauliflower grown using a NH_4_:NO_3_ 25:75 molar ratio provided a contribution of magnesium of almost 15% of the Dietary Reference Value, while in all other cases the contribution was of about 9.3–11.6% (Table 4). The contribution of iron ranged from 4.5% to 7.5% of the DRV. With respect to sodium, the contribution was very low, considering that the highest content was found in micro cauliflower that was grown using a NH_4_:NO_3_ 25:75 molar ratio, which translates into a contribution of about 0.9%. Among other mineral elements, the nutrient contributions of *Brassica* microgreens were 12.8–19%, 5.0–7.8%, 6.8–11.4%, 1.0–1.8% and 9.8–18.9% of the DRV, respectively for calcium, potassium, zinc, copper and manganese (Table 5).

**Figure 4:**
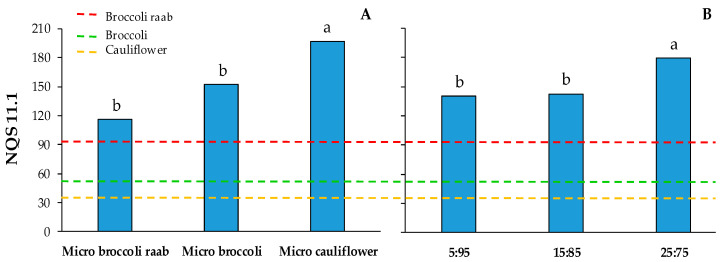
Main effects of genotype (micro broccoli raab, micro broccoli and micro cauliflower—(**A**)) and NH_4_:NO_3_ molar ratio of the nutrient solution (5:95, 15:85, and 25:75—(**B**)) on Nutrient Quality Score (NQS) 11.1 of *Brassica* microgreens. Significance: *p* ≤ 0.05 (**A**); *p* ≤ 0.05 (**B**). For each main effect, different letters indicate that mean values are significantly different (*p* = 0.05). The horizontal lines indicate the NQS 11.1 of mature broccoli raab, broccoli and cauliflower.

The effects of genotypes and the NH_4_:NO_3_ molar ratio on NQS 11.1 are reported in Figure 4, whereas the interaction between genotypes and NH_4_:NO_3_ molar ratio was not significant (Supplementary Table S1). Independent of the NH_4_:NO_3_ molar ratio, the Nutrient Quality Score (NQS) 11.1 was highest in micro cauliflower, which showed a score that was 47% higher in comparison to those of micro broccoli raab and micro broccoli (Figure 4A). At the same time, by using NH_4_:NO_3_ 25:75 molar ratio, the NQS 11.1 was 27% higher than other molar ratios (Figure 4B). In all cases the NQSs 11.1 of *Brassica* microgreens were higher than those of mature broccoli raab, broccoli and cauliflower (Figure 4).

## 4. Discussion

In this study, the nutritional value of three *Brassica* microgreens was assessed by using a Nutrient Quality Score in order to better evaluate the contributions of macro- and micronutrients in the diet. For microgreen growth, we used a nutrient solution with ion concentrations similar to those reported by Hoagland and Arnon [25], but at half strength and having three different NH_4_:NO_3_ ratios (%): 5:95, 15:85 and 25:75. This decision was based on the remark that a moderate concentration of N-NH_4_ in the nutrient solution improved the performance of *B. pekinensis* microgreens in comparison to the use of only N-NO_3_ [43].

As for the nutrient content, we found that, independent from the three NH_4_:NO_3_ ratios, the contents of fiber, β-carotene, α-tocopherol, calcium, potassium, zinc, copper and manganese in the microgreens were not significantly different among the three *Brassica* genotypes. At the same time, we observed that micro cauliflower grown with a NH_4_:NO_3_ 25:75 molar ratio showed a higher content of protein, manganese and sodium than the other samples (Table 2 and Table 3). The ANOVA for protein magnesium and sodium content was confirmed by PCA. At the same time, PCA also revealed other positive correlations, but ANOVA did not allow us to observe differences that could be confirmed by PCA.

From a nutritional point of view, it is interesting to report that in a study aimed at evaluating the mineral content of 30 varieties of *Brassicaceae* microgreens, Xiao et al. [24] concluded that these vegetable products can be considered to be good sources of mineral elements (such as calcium, potassium, iron and zinc) in a balanced human diet. In our study, we observed a higher average content of calcium, manganese and zinc, a lower content of sodium and copper and a similar content of magnesium, potassium and iron in comparison with data reported by these authors. Therefore, our results suggest that the mineral content in *Brassicaceae* microgreens can vary among different genotypes; however, this also depends on the nutrient solution that is used.

With respect to protein content, our results are in agreement with other studies. Effectively, Renna et al. [17] found an average content of 1.8–1.9 g 100 g^−1^ FW in micro lettuce and micro chicory, while among 10 culinary microgreens, Ghoor et al. [14] found a protein content ranging from 1.8 to 4.4 g 100 g^−1^ FW. On the other hand, although Ghoor et al. [14] indicated a fiber content ranging from 1.4 to 4.3 g 100 g^−1^, we observed an average fiber content similar to those reported in other studies [9,17]. Therefore, results from the present study confirm that the protein and fiber content in microgreens can vary depending on the genotype, also suggesting that the growing conditions have an influence on these nutritional traits.

The quantification of nutrients in microgreens is surely useful for evaluating the potential effects of experimental factors, as well as for carrying out comparisons with other vegetable products. On the other hand, nutrient quantification alone is not sufficient for evaluating nutrients’ relative contributions to the diet. Therefore, we calculated the percent of nutritional contribution for each nutrient per serving size of microgreens (Table 4 and Table 5). According to the U.S. Food and Drug Administration, a food product can be considered a ‘good source’ of a specific nutrient if a serving size contains 10% to 19% of the DRV for that nutrient, while the term ‘excellent source’ can be used if a serving size contains 20% or more of the DRV [44]. From these requirements, our results indicate that in all cases *Brassica* microgreens can be considered as an excellent source of vitamins A and E (Table 4). Our results are in agreement with Ghoora et al. [14], who describe 10 culinary microgreens as an excellent source of these vitamins, since the percentages of the nutritional contribution of vitamins A and E were 23.3–71.8 and 28.5–332, respectively.

With respect to mineral elements, calcium and manganese displayed a higher percent of nutritional contribution than other elements, suggesting that *Brassica* microgreens can be considered as a good source of these elements (Table 5). Cauliflower grown using a NH_4_:NO_3_ 25:75 molar ratio can certainly be considered a good source of magnesium, while in all other cases the nutritional contribution of magnesium (on average 10%) is borderline when considering *Brassica* microgreens as a good source of magnesium. For all other elements, the percent of nutritional contribution is not high enough to consider *Brassica* microgreens as a good source (Table 5). Our results are in agreement with Ghoora et al. [14], with the exception of those for calcium, for which Ghoora et al. [14] found nutritional contribution values lower than 10% for all microgreens genotypes.

In order to evaluate the summative contribution of all quantified nutrients, we developed the NQS 11.1 as a useful tool for evaluating the overall nutritional quality of *Brassica* microgreens. By using a NQS, it is possible to go beyond the evaluation of a food product on the basis of one nutrient at a time, while avoiding defining any food as ‘good’ or not, in absolute terms. The setup of our NQS was derived from the well-known concept of nutrient density, which is the basis of several indexes and scores in the literature [2,45,46,47,48]. On the other hand, although the nutrient density indexes and scores are usually calculated for each food item per 100 kcal, we established the NQS 11.1 in relation to the summative contribution of nutrients in a serving size, according to Ghoora et al. [14]. We did this considering that our NQS could enable consumers to compare and choose from several plant-derived ingredients and foods, on the basis of overall nutritional quality of the same serving size.

In the present study, we observed a higher NQS 11.1 in micro cauliflower than micro broccoli raab and micro broccoli (Figure 4A). Likewise, Ghoora et al. [14] found a variability of the NQS among different species, with the highest value for micro radish and the lowest value for micro fenugreek. Considering that both broccoli and cauliflower belong to the same species (*Brassica oleracea* L.), our results highlight the effectiveness of the NQS 11.1 in distinguishing differences in terms of overall nutritional quality, not only among different species, but also comparing genotypes that are not very different from a genetic point of view. It is also important to highlight that, for the first time, a NQS score was used to evaluate an important aspect of the cultivation of microgreens, that is, the influence of different nutrient solutions. To this end, we observed significant differences among the three nutrient solutions, with the highest NQS 11.1 achieved using the NH_4_:NO_3_ 25:75 molar ratio (Figure 4B). In this context, it is important to highlight that by using high amounts of ammonium as a form of nitrogen, plants can exhibit NH_4_^+^ toxicity symptoms, due to the pH decrease in the nutrient solution as a consequence of hydrogen ion release from the roots [49,50]. On the other hand, it has been reported that supplying a portion of nitrogen as ammonium in the nutrient solution can improve the qualitative traits of leafy vegetables [43,50]. This could be due to different physiological mechanisms, such as the regulation of root architecture and photosynthesis [43]. In agreement, our results suggest that the NH_4_:NO_3_ 25:75 molar ratio may be the best for *Brassica* microgreens, also considering the short growing cycle and the optimal pH range of the used nutrient solution.

To the best of our knowledge, the NQS on micro broccoli raab, micro broccoli and micro cauliflower was carried out for the first time in the present study. Thus, from a nutritional point of view, it was interesting to compare the NQS 11.1 of our microgreens with the average NQS 11.1 of mature broccoli, mature cauliflower and mature broccoli raab. To this end, we decided to use data from the United State Department of Agriculture [32,33,34], since this has been the primary food composition data resource in the United States for decades, providing a comprehensive list of nutrient and food component values that are derived from analyses, calculations and published literature. It is an important premise that mature broccoli raab showed the highest NQS 11.1, this being 183% and 72% higher than mature cauliflower and mature broccoli, respectively (Figure 4). This is because mature broccoli raab showed the highest values in the percent of nutritional contribution for some nutrients, such as vitamins A and E, calcium, iron and manganese, compared to mature cauliflower and mature broccoli (data not shown). On the other hand, we observed that all *Brassica* microgreens resulted in a higher NQS 11.1 than their mature counterparts, highlighting that micro cauliflower’s score was about six times higher than that of mature cauliflower (Figure 4A). The highest NQS 11.1 in micro cauliflower is primarily due to the percentage nutritional contributions of vitamins A and E (Table 4). These results are in agreement with Xiao et al. [51], who found that micro broccoli and micro cauliflower had higher concentrations of carotenoids than their mature counterparts. According to Podsedek [52], the carotenoid concentration in mature cauliflower can be considered very low. At the same time, the higher amount of carotenoids in micro cauliflower could be due to the essential activity in microgreens’ leaf tissues associated with photosynthesis, but for this reason, they are negligible or absent in the heads of cauliflower [51].

Apart from the main effect of genotype on the NQS 11.1, we observed that, independent from the NH_4_:NO_3_ molar ratio of the nutrient solution, the NQS 11.1 of *Brassica* microgreens was higher in comparison to the mature counterpart (Figure 4B). In a study aimed at evaluating the amount of ascorbic acid, carotenoids, phylloquinone and tocopherols in 25 genotypes of microgreens, Xiao et al. [8] found that microgreens showed higher nutritional values in comparison to their mature counterparts. Other authors reported higher mineral contents in micro broccoli and micro lettuce, compared to their mature leaves [22,53]. By using a Nutrient Quality Score similar to that developed in this study, Ghoora et al. [14] found that 10 species of microgreens showed scores 2.0–3.5 times higher than mature spinach. Our results confirm that microgreens can be considered as nutrient-rich vegetables, which are able to improve dietary patterns more effectively than mature vegetables, also highlighting that the use of NQS 11.1 can be very useful for assessing the overall nutritional value.

## 5. Conclusions

This study highlights the helpful use of NQS 11.1 for evaluating the overall nutritional quality of microgreens, instead of simply quantifying nutrient content to evaluate the variations of a single nutrient among different genotypes. Furthermore, for the first time, a NQS was used not only to compare different genotypes of microgreens, but also to assess the effects of growing conditions on nutritional quality. Results show that the micro broccoli raab, micro broccoli and micro cauliflower in this study can be considered as nutrient-rich vegetables that are able to improve dietary patterns more effectively than their mature counterparts. Therefore, the wide spread of microgreens as food ingredients could be a health-promoting strategy for better meeting the dietary reference intake requirements of the essential elements that are beneficial to human health. Moreover, the results of the present study highlight the effectiveness of the NQS 11.1 in distinguishing differences in terms of overall nutritional quality, not only among different growing conditions, but also in comparing genotypes that are not very different from a genetic point of view. By virtue of the large number of different nutrients that it considers simultaneously, NQS 11.1 could be applied to any food product or recipe in order to derive a better overview of nutritional quality, as well as in the case of the nutrition label found on packaged foods. Future research activities may be aimed at the evaluation of the overall nutritional value of other microgreen-based products, and also at determining the potential that other plant-derived ingredients have in improving dietary patterns and fighting hidden hunger.

## Figures and Tables

**Table 1:** Dietary Reference Values (DRV) for selected nutrients used in developing the NQS 11.1.

Nutrient	DRV	References
Protein (g)	62	[35]
Fiber (g)	25	[3]
Vitamin A (μg RAE) *	750	[4]
Vitamin E (mg)	13	[5]
Ca (mg)	950	[6]
K (mg)	3500	[36]
Mg (mg)	350	[37]
Fe (mg)	11	[38]
Zn (mg)	11.7	[39]
Cu (mg)	1.6	[40]
Mn (mg)	3	[41]
Na (mg)	2000	[42]

* The retinol activity equivalency (μg RAE) ratio for β-carotene from food was estimated to be 12:1 [4].

**Table 2:** Content of protein, fiber, β-carotene and α-tocopherol in *Brassica* microgreens grown by using a nutrient solution with three NH_4_:NO_3_ molar ratios.

Genotype	NH_4_:NO_3_ Molar Ratio	Value	Protein	Fiber	β-Carotene	α-Tocopherol
			g 100 g^−1^ FW		mg 100 g^−1^ FW	
Micro broccoli raab	5:95	Mean	2.34 b	0.49	4.05	1.84
		SD	0.28	0.05	1.82	0.58
	15:85	Mean	2.29 b	0.47	3.54	1.81
		SD	0.06	0.02	0.47	0.64
	25:75	Mean	2.41 b	0.45	3.27	2.63
		SD	0.31	0.02	0.94	0.63
Micro broccoli	5:95	Mean	2.19 b	0.56	4.61	6.43
		SD	0.10	0.02	1.14	0.20
	15:85	Mean	2.41 b	0.44	4.92	6.37
		SD	0.30	0.07	2.24	0.50
	25:75	Mean	2.41 b	0.61	6.51	6.30
		SD	0.51	0.01	0.04	0.11
Micro cauliflower	5:95	Mean	2.44 b	0.46	4.32	6.32
		SD	0.23	0.13	0.94	6.59
	15:85	Mean	2.50 b	0.62	5.33	8.37
		SD	0.32	0.19	0.88	5.41
	25:75	Mean	4.20 a	0.89	9.08	14.93
		SD	1.14	0.26	1.63	10.28
Significance						
Genotype (G)			NS	*	**	*
Molar ratio (M)			NS	NS	**	NS
G × M			*	NS	NS	NS

Significance: ** significant for *p* ≤ 0.01; * significant for *p* ≤ 0.05; NS, not significant. Mean values (±standard deviation—SD) in each column followed by different letters are significantly different, according to LSD test (*p* = 0.05).

**Table 3:** Elements content in *Brassica* microgreens grown by using a nutrient solution with three NH_4_:NO_3_ molar ratios.

Genotype	NH_4_:NO_3_ Molar Ratio	Value	Ca	K	Mg	Na	Zn	Fe	Cu	Mn
			mg 100 g^−1^ FW				μg 100 g^−1^ FW			
Micro broccoli raab	5:95	Mean	189.6	254.7	47.7 b	11.3 bcd	1711	774 ab	31.0	669
		SD	22.8	60.7	4.9	1.6	150	57	0.9	64
	15:85	Mean	166.5	268.5	41.7 b	9 cd	1591	665 ab	30.9	465
		SD	22.5	32.1	6.0	1.8	216	154	7.2	81
	25:75	Mean	149.9	253.0	39.3 b	8.1 d	1737	584 b	34.0	427
		SD	18.4	41.8	4.8	1.0	238	24	5.4	50
Micro broccoli	5:95	Mean	157.9	216.3	38.2 b	12.3 bc	1021	594 b	27.3	503
		SD	4.9	10.4	1.7	1.0	48	71	0.6	19
	15:85	Mean	165.4	258.4	40.5 b	11.6 bcd	1125	721 ab	26.4	424
		SD	20.2	52.7	5.1	1.8	286	149	4.5	57
	25:75	Mean	143.6	251.8	38.1 b	11.5 bcd	1112	596 b	25.5	380
		SD	23.8	80.3	5.50	0.6	286	81	3.0	41
Micro cauliflower	5:95	Mean	162.3	210.4	43.3 b	13.1 b	933	679 ab	19.2	439
		SD	4.1	13.6	2.4	0.5	17	13	3.4	49
	15:85	Mean	153.6	207.3	40.7 b	10.7 bcd	1002	632 b	19.0	346
		SD	25.4	64.6	4.3	1.1	224	78	2.7	40
	25:75	Mean	212.9	322.0	60.1 a	18.0 a	1566	879 a	28.8	481
		SD	47.4	89.4	11.2	4.4	487	197	10.3	103
Significance										
Genotype (G)			NS	NS	*	**	**	NS	**	*
Molar ratio (M)			NS	NS	NS	NS	NS	NS	NS	*
G × M			NS	NS	*	*	NS	*	NS	NS

Significance: ** significant for *p* ≤ 0.01; * significant for *p* ≤ 0.05; NS, not significant. Mean values (±standard deviation—SD) within each column followed by different letters are significantly different, according to LSD test (*p* = 0.05).

**Table 4:** Percent of nutritional contribution (± standard deviation) of protein, fiber, vitamin A (as β-carotene) and vitamin E (as α-tocopherol) per serving size of *Brassica* microgreens based on the Dietary Reference Values (DRV).

Genotype	NH_4_:NO_3_ Molar Ratio	Protein	Fiber	Vitamin A	Vitamin E
Micro broccoli raab	5:95	3.21 ± 0.38	1.68 ± 0.17	38.3 ± 17.2	12.1 ± 3.8
	15:85	3.14 ± 0.08	1.60 ± 0.06	33.5 ± 4.4	11.8 ± 4.2
	25:75	3.31 ± 0.43	1.55 ± 0.06	30.9 ± 8.9	15.5 ± 4.1
Micro broccoli	5:95	3.00 ± 0.14	1.89 ± 0.03	43.5 ± 10.8	42.1 ± 1.3
	15:85	3.30 ± 0.42	1.50 ± 0.23	46.5 ± 21.2	41.8 ± 3.3
	25:75	3.31 ± 0.71	2.07 ± 0.05	61.5 ± 0.4	41.2 ± 0.7
Micro cauliflower	5:95	3.34 ± 0.31	1.56 ± 0.44	40.8 ± 8.9	41.32 ± 43.1
	15:85	3.43 ± 0.43	2.11 ± 0.65	50.3 ± 8.4	57.1 ± 35.4
	25:75	5.76 ± 1.56	3.30 ± 0.90	85.7 ± 15.4	97.6 ± 47.2

**Table 5:** Percent of nutritional contribution (± standard deviation) of mineral elements per serving size of *Brassica* microgreens based on the Dietary Reference Values.

Genotype	NH_4_:NO_3_ Molar Ratio	Ca	K	Mg	Na	Zn	Fe	Cu	Mn
Micro broccoli raab	5:95	17.0 ± 2.0	6.2 ± 1.5	11.6 ± 1.2	0.5 ± 0.1	12.4 ± 1.1	6.0 ± 0.4	1.6 ± 0.1	18.9 ± 1.8
	15:85	14.9 ± 2.0	6.5 ± 0.8	10.1 ± 1.5	0.4 ± 0.1	11.6 ± 1.6	5.1 ± 1.2	1.6 ± 0.4	13.2 ± 2.3
	25:75	13.4 ± 1.6	6.1 ± 0.8	9.5 ± 1.5	0.3 ± 0.1	12.6 ± 1.7	4.5 ± 0.2	1.8 ± 0.3	12.1 ± 1.4
Micro broccoli	5:95	14.1 ± 0.4	5.2 ± 0.3	9.3 ± 0.4	0.5 ± 0.1	7.4 ± 0.4	4.6 ± 0.5	1.5 ± 0.1	14.2 ± 0.5
	15:85	14.8 ± 1.8	6.3 ± 1.3	9.8 ± 1.2	0.5 ± 0.1	8.2 ± 2.1	5.6 ± 1.2	1.4 ± 0.2	12.0 ± 1.6
	25:75	12.8 ± 2.1	6.1 ± 1.9	9.2 ± 1.3	0.5 ± 0.1	8.1 ± 2.1	4.6 ± 0.6	1.4 ± 0.2	10.8 ± 1.2
Micro cauliflower	5:95	14.5 ± 0.4	5.1 ± 0.3	10.5 ± 0.6	0.6 ± 0.1	6.8 ± 0.1	5.2 ± 0.1	1.0 ± 0.2	12.4 ± 1.4
	15:85	13.7 ± 2.3	5.1 ± 1.6	9.9 ± 1.3	0.5 ± 0.1	7.3 ± 1.6	5.0 ± 0.6	1.0 ± 0.1	9.8 ± 1.1
	25:75	19.1 ± 4.2	7.8 ± 2.2	14.6 ± 3.3	0.9 ± 0.2	11.4 ± 3.5	7.5 ± 1.5	1.5 ± 0.5	13.6 ± 2.9

## Supplementary Materials

The following are available online at https://www.mdpi.com/2304-8158/9/9/1226/s1, Table S1: Nutrient Quality Score (NQS) 11.1 (value ± standard deviation – SD) of three *Brassica* microgreens (micro broccoli raab, micro broccoli and micro cauliflower) grown by using three NH_4_:NO_3_ molar ratio (5:95, 15:85, and 25:75) nutrient solutions.
