# Supplementary material for: The Use of a Nutrient Quality Score is Effective to Assess the Overall Nutritional Value of Three Brassica Microgreens
Source: Foods. 2020 Sep 2;9(9):1226. doi: 10.3390/foods9091226 (PMC7555376; doi:10.3390/foods9091226)
Supplement: Supplementary file 1 [file foods-09-01226-s001.pdf]

Table S1. Nutrient Quality Score (NQS) 11.1 (value ± standard deviation – SD) of three *Brassica* microgreens (micro broccoli raab, micro broccoli and micro cauliflower) grown by using three NH_4:NO_3 molar ratio (5:95, 15:85, and 25:75) nutrient solutions.

**Genotype**	**NH_4:NO_3 molar ratio**	**Value**	**NQS 11.1**
Micro broccoli raab	5:95	Mean	128.5
		SD	13.4
	15:85	Mean	112.7
		SD	6.3
	25:75	Mean	111.0
		SD	20.1
Micro broccoli	5:95	Mean	146.3
		SD	10.1
	15:85	Mean	150.6
		SD	28.2
	25:75	Mean	160.6
		SD	10.3
Micro cauliflower	5:95	Mean	142.1
		SD	55.4
	15:85	Mean	164.2
		SD	36.2
	25:75	Mean	266.1
		SD	87.6
Significance			NS

Significance: NS, not significant.
